# Fear of cancer levels of women who applied cervical cancer screening: a cross-sectional study of the effects of health beliefs related to cervical cancer and Pap-Smear test

**DOI:** 10.1007/s10552-025-02008-0

**Published:** 2025-05-25

**Authors:** Ayse Deliktas Demirci, Melek Avci, Kamile Kabukcuoglu

**Affiliations:** 1 Faculty of Nursing, Department of Obstetrics & Gynaecological Nursing, Akdeniz University, Dumlupınar Bulvarı, 07058 Antalya, Turkey; 2 Health Services Vocational School, Akdeniz University, Antalya, Turkey

**Keywords:** Fear of cancer, Cervical cancer, Pap Smear test, Screening behaviors, Culture, Meaning of cancer

## Abstract

**Background:**

Excessive fear of cancer can be more harmful than the disease itself. Although it is important to address and evaluate this fear, there is a lack of studies providing intervention strategies to effectively reduce cancer-related anxiety.

**Aim:**

This study aimed to evaluate how health beliefs about cervical cancer and the Pap Smear test influence cancer fear among women participating in a cervical cancer screening program.

**Methods:**

This was a cross-sectional study. Data were gathered from 210 women who visited a healthy life center for cervical cancer screening. The data collection tools included a personal form, The Health Belief Model Scale for Cervical Cancer and the Pap Smear Test, and the Cancer Worry Scale. Statistical analyses, such as *T*-tests, ANOVA, and hierarchical linear multiple regression, were conducted using the IBM SPSS software version 23.0.

**Findings:**

The regression model assessing the impact of the benefits of pap smear tests on cancer worry was not statistically significant (*p* > 0.05). However, when barriers to the pap smear test were added, there was a statistically significant change in *R*^2^ by 0.031 (*p* = 0.012), and including the perceived seriousness of cervical cancer further increased the *R*^2^ value by 0.126, which was statistically significant (*p* = 0.000). Adding susceptibility to cervical cancer and health motivation individually to the model did not lead to a statistically significant change in *R*^2^ (*p* = 0.060, *p* = 0.655, respectively). The final model, which included all concepts from The Health Belief Model Scale for Cervical Cancer and the Pap Smear Test, accounted for 15.6% of the variance in cancer worry based on independent variables. Additionally, employed women (*t* = − 2.461, *p* = 0.015) and nuclear families (*t* = − 2.554, *p* = 0.011) had significantly lower cancer anxiety scores.

**Conclusion:**

The study indicated that perceived severity and barriers might heighten fear of cancer. These perceptions could be influenced by social environment, media, and language issues related to the meanings of “cancer”. Therefore, oncological care should incorporate culturally sensitive approaches to assess and reduce fear of cancer. Collaboration with public health managers is necessary to develop campaigns that address this issue without inciting fear.

## Introduction

Fear of cancer, or carcinophobia, is an emotional reaction characterized by anxiety related to cancer risks, including diagnosis, treatment, and potential negative outcomes. This fear is deeply rooted in society, with cancer viewed as a severe, unpredictable, and potentially fatal illness that triggers distressing thoughts about suffering and mortality [1]. Fear of cancer includes concerns about pain, loss of autonomy, social isolation, emotional turmoil, and death [2]. Despite progress in cancer detection and treatment, research shows that a considerable portion of the population—between one-third and one-half of nations such as the United States and the United Kingdom—fears cancer more than any other disease [3, 4]. Approximately 5–10% of individuals experience extreme concern, significantly affecting their mental health and daily functioning [5]. Intense fear of cancer can be harmful and counterproductive, affecting one’s willingness to participate in cancer screenings, use healthcare services, and follow through with recommended follow-ups, thereby impeding cancer prevention efforts [6]. This fear can be ongoing and distressing, influencing the daily lives and well-being of patients and their families [7]. Moreover, this may result in unnecessary medical tests [8], which can be expensive and potentially harmful [9]. Given the progress in cancer diagnostics and treatment, excessive fear of cancer might be more damaging than the disease itself. Consequently, it is important to address, anticipate, and assess the excessive fear of cancer [7].

Globally, cervical cancer stands as the most common gynecological malignancy and the sole gynecological cancer integrated into national screening programs with established protocols [10, 11]. Although pap-smear tests play a crucial role in early detection, screening rates remain inadequate, particularly in developing and underdeveloped nations [12]. Fear is identified as a primary barrier to cervical cancer screening, which leads to delays in early detection and treatment [13]. The fear of cervical cancer serves as both a motivating factor [14, 15], and deterrent for screening participation due to overwhelming anxiety [16, 17]. Research frequently addresses fear of cancer, examining the factors influencing it [18–20] and participation in screening programs [16, 1, 4]. However, there is a scarcity of studies offering interventional recommendations that may effectively reduce fear of cancer.

The Health Belief Model (HBM) is a key framework for understanding health behaviors, often used to boost cervical cancer screening participation [21, 22]. Developed by Hochbaum, Leventhal, Kegeles, and Rosenstock in the 1950s, the model explains how perceptions shape health choices. The HBM includes four elements: perceived susceptibility, severity, benefits, and barriers [23]. Shen and Acevedo Callejas [24] noted fear of cancer is influenced by threat assessments of the disease and treatment side effects. Risk estimates are affected by emotional responses and threat appraisals. Since fear of cancer is impacted by threat assessments and emotional reactions, HBM-based interventions could help alleviate cancer-related fears. More studies are needed to evaluate the effect of HBM-based interventions on fear of cancer and develop strategies to overcome fear-driven avoidance of cervical cancer screening. Therefore, the present study aimed to assess the impact of health beliefs related with cervical cancer and the pap smear test on fear of cancer among women who applied cervical cancer screening program.

### Hypothesis

**H1:** Health belief levels of women applying for cervical cancer screening program has a role in reducing fear of cancer.

## Material and methods

### Design

This study was conducted in a cross-sectional research design. The Strengthening the Reporting of Observational Studies in Epidemiology (STROBE) checklist for cross-sectional studies [25] was followed while conducting the study.

### Setting

The present study was conducted in Türkiye between April 2023 and August 2023. Data were collected from women who applied to a healthy life center, which is the largest center in the southern city of Türkiye. This center is constructed by the National Health Ministry, which offers free charges for every woman to apply for a cervical cancer screening unit.

### Sampling and recruitment

The study utilized convenience sampling was used to enrol subjects from a healthy life center’s cancer screening division. The study eligibility requirements were as follows: (1) enrollment in the cervical cancer screening program, (2) age ≥ 18 years, and (3) voluntary participation. Women who were unable to communicate in Turkish were also excluded. The G-Power program version 3.1.9.7 (Heinrich Heine University, Dusseldorf, Germany) was utilized to determine the necessary sample size. For a two-tailed analysis comparing two independent means, a type 1 error rate (a) of 0.05 and 95% study power were used. Given the absence of prior research on cancer fear levels among women in Türkiye’s cervical cancer program, a Cohen’s medium effect size of 0.5 was employed, resulting in a sample size of 210 women. A total of 225 women were asked to participate; however, only 210 women completed the survey (*N* = 210, response rate: 93.3%), while 15 women refused to answer the questions in the survey. A total of 210 recruited participants completed the study. According to Kendall’s guidelines, the recommended sample size should be to 5–10 times the number of variables [26]. With five variables in the final regression analysis, 210 participants were deemed sufficient for the hierarchical linear multiple regression analysis.

### Data collection

Prior to data collection, the researchers (MA, ADD) provided informed consent at the center. The participants were interviewed in person, with an average interview duration of 20 min.

### Instruments

The data collection tools used during data collection are as follows;

*The personal form* provides information about participants’ socio-demographic and cancer screening processes,

#### The health belief model scale for cervical cancer and the Pap Smear test

Güvenç et al. [27] developed and validated the Health Belief Model Scale for Cervical Cancer and the Pap Smear Test, drawing from Health Belief Theory. This instrument employs a 5-point Likert scale with 35 items. The overall scale’s Cronbach Alpha reliability coefficient ranges from 0.62 to 0.86, while its subscales’ coefficients span from 0.79 to 0.87. Scale evaluation is evaluated as “strongly disagree” one point, “strongly agree” five points. The scale comprises five sub-dimensions, each evaluated independently without being combined into a single score. These sub-dimensions include: “Pap smear benefits/health motivation” (Eight items, 8–40 points), “Pap smear barriers” (14 items, 14–70 points), “Seriousness” (Seven items, 7–35 points), “Susceptibility” (Three items, 3–15 points), and “Health motivation” (Three items, 3–15 points). All sub-dimensions, except for the perception of barriers, showed a positive correlation with Pap Smear screening behavior. Cronbach’s *α* values were calculated for each sub-dimension: sensitivity (0.81), care-seriousness (0.78), benefit-motivation (0.92), health motivation (0.68), and barriers (0.78).

#### Cancer worry scale

Custers et al. [28] developed this instrument to evaluate anxiety levels related to cancer in women. Üner and Körükçü [20] conducted a study to validate its reliability in Türkiye. The instrument comprises eight items, with responses rated on a 4-point Likert scale. Scores on the scale range from 8 to 32, with higher scores indicating more frequent cancer-related anxiety. The Turkish version of the scale had a Cronbach’s alpha value of 0.77. The present study Cronbach’s *α* values was calculated as 0.83.

### Data analysis

Individual characteristics were examined using descriptive statistics. Frequency and percentage were applied to describe categorical variables in the socio-demographic data, while continuous variables were characterized by their mean and standard deviation (SD). The internal consistency of each measurement was evaluated using Cronbach’s alpha (*α*). Several assumptions were tested before implementing the regression model, including normality, multicollinearity, linearity, and homoscedasticity. Normal distribution of the data was confirmed by a Kolmogorov–Smirnov/Shapiro–Wilk test *p*-value of ≥ 0.05, skewness ranging from − 2 to + 2, and kurtosis between − 2 and + 2. The data also demonstrated linearity with a correlation coefficient of < 0.25. Multicollinearity was not a concern, as values surpassed 0.85 and VIF values remained below 5.

A hierarchical linear multiple regression analysis was conducted to investigate the factors affecting cancer fear (using the entry technique). The study incorporated variables in a stepwise manner: benefits of Pap smear tests in Step 1, barriers to pap smear test in Step 2, perceived seriousness of cervical cancer in Step 3, susceptibility to cervical cancer in Step 4, and health motivation in Step 5. The study employed a significance level of *p* 0.05. Data analysis was performed using SPSS 23 software (SPSS Inc., Chicago, IL, USA).

### Ethical considerations

Each participant was informed about the study’s purpose and the nature of the interviews. Written consent was obtained from all participants. The research was conducted in accordance with the guiding principles of the Declaration of Helsinki. The study received approval from the regional Ethics Committee of Akdeniz University in Antalya, Türkiye (Date: 22.12.2021/No: KAEK-897).

### Fındings

The majority of the women (*n* = 126, 60%) were found to be in the younger age group (30–40 years). Regarding educational attainment, primary education was the most common (*n* = 91, 43.3%), followed by bachelor’s degree or higher (*n* = 77, 33.3%). It was determined that most women were unemployed (*n* = 135, 64.3%), perceived their economic income as equal to their expenses (*n* = 121, 57.65%), and resided in urban areas (*n* = 202, 96.2%). Additionally, most participants were married (*n* = 194, 92.4%), belonged to a nuclear family structure (*n* = 185, 88.1%), and had children (*n* = 196, 93.3%).

A significant proportion of women reported that they had never undergone cervical cancer screening before and were applying for the test for the first time (*n* = 128, 61%). Among those who had previously undergone screening, the percentage who did so regularly was recorded, with the primary reason for regular screening being routine health check-ups (*n* = 20, 47.6%). Conversely, those who did not undergo regular screening cited a lack of awareness about cancer screening as the main reason (*n* = 108, 84.4%). Healthcare professionals were the most common source of information regarding screening (*n* = 181, 86.2%).

The mean scores related to women’s perceptions of cervical cancer screening were as follows: The benefits and health motivation related to Pap smears scored 33.74 ± 5.51 out of a possible 40 points. Barriers to Pap smears were rated at 30.63 ± 8.22 out of a maximum of 70 points. The seriousness score was 21.70 ± 5.96 out of 35 points, while susceptibility was measured at 7.40 ± 2.27 out of 15 points. Health motivation achieved a score of 10.95 ± 2.43 out of 15 points. The overall cancer worry score was 15.16 ± 4.57 out of a total of 32 points. When examining the factors influencing total cancer worry scores, it was observed that employed women (*t* = − 2.461, *p* = 0.015) and those from nuclear families (*t* = − 2.554, *p* = 0.011) had significantly lower cancer anxiety scores (See Table 1).

**Table 1:** Characteristics of women (*N* = 210)

Characteristics	*n* (%)	M ± SD	Cancer worry scale	
			M ± SD	t/F/r (*p*)
Age				
Early middle age (30–40)	126 (60)		15.57 ± 4.75	1.147, *p* = 0.253
Middle age (40–60)	84 (40)		14.73 ± 4.75	
Educational status				
Primary school	91 (43.3)		15.69 ± 0.49	1.056, *p* = 0.400
High school	49 (23.3)		15.56 ± 0.72	
Bachelor’s degree and above	70 (33.3)		14.22 ± 0.49	
Employment status				
Yes	75 (35.7)		14.37 ± 4.27	− 2.461, *p* = 0.015*
No	135 (64.3)		16.05 ± 4.97	
Marital status				
Married	194 (92.4)		15.38 ± 4.74	− 0.692, p = 0.490
Single	16 (7.6)		16.25 ± 5.45	
Family type				
Nuclear	185 (88.1)		15.14 ± 4.85	− 2.554, p = 0.011*
Extended	25 (11.9)		17.72 ± 3.61	
Having children				
Yes	196 (93.3)		15.57 ± 4.85	1.350, *p* = 0.179
No	14 (6.7)		13.78 ± 3.57	
Place of residence				
Center	202 (96.2)		15.43 ± 4.77	− 0.254, *p* = 0.800
District	8 (3.8)		15.87 ± 5.56	
Perceived income level				
Good	40 (19)		15.92 ± 0.73	1.286, *p* = 0.185
Medium	121 (57.6)		14.91 ± 0.41	
Poor	49 (23.3)		15.17 ± 0.67	
Having been screened before				
Yes	82 (39)		15.68 ± 5.00	0.557, *p* = 0.578
No	128 (61)		15.30 ± 4.66	
Screening frequency of before (*n* = 82)				
Regular	42 (51.2)		15.82 ± 4.93	1.588, *p* = 0.115
Irregular	40 (48.8)		14.50 ± 4.08	
Reason for regular screening of screened before (*n* = 42)				
Check-up	20 (47.6)		14.88 ± 0.49	1.019, *p* = 0.443
Cancer anxiety	14 (33.3)		15.12 ± 1.34	
Diagnosis	8 (19.1)		14.35 ± 0.88	
Reason for not having screening (*n* = 128)				
Not awareness	108 (84.4)		15.86 ± 4.85	− 1.611, 0.111
Feeling ashamed	20 (15.6)		18.54 ± 7.00	
Getting information for screening				
Healthcare professionals	181 (86.2)		15.08 ± 0.34	1.124, *p* = 0.325
Social environment	21 (10)		16.46 ± 1.45	
Media	8 (3.8)		14.12 ± 0.39	
The health belief model scale for cervical cancer and the pap smear test				
Benefits of pap smear tests		33.74 ± 5.51 (13–40)		
Barriers to pap smear test		30.63 ± 8.22 (14–58)		
Perceived seriousness of cervical cancer		21.70 ± 5.96 (7–35)		
Health motivation		10.95 ± 2.43 (4–15)		
Susceptibility to cervical cancer		7.40 ± 2.27 (3–13)		
Cancer worry scale		15.17 ± 4.57 (2–29		

*t* t test for independent groups, *M* Mean, *SD* Standard deviation, F:ANOVA.*p significance value *p* < 0.05

In assessing the impact of the Health Belief Model Scale for Cervical Cancer and the Pap Smear Test on cancer worry scores, the regression model evaluating the effect of benefits of pap smear tests on cancer worry was not statistically significant (*p* > 0.05). However, regression models analyzing the effects of all other sub-dimensions of the Health Belief Model Scale for Cervical Cancer and the Pap Smear Test on cancer worry were statistically significant (*p* ≤ 0.05) (Table 2).

**Table 2:** ANOVA

ANOVA^a^						
Model		Sum of squares	df	Mean square	F	Sig
1	Regression	18.869	1	18.869	0.902	0.343^b^
	Residual	4265.519	204	20.909		
	Total	4284.388	205			
2	Regression	149.692	2	74.846	3.675	0.027^c^
	Residual	4134.696	203	20.368		
	Total	4284.388	205			
3	Regression	688.908	3	229.636	12.901	0.000^d^
	Residual	3595.480	202	17.799		
	Total	4284.388	205			
4	Regression	751.974	4	187.994	10.697	0.000^e^
	Residual	3532.414	201	17.574		
	Total	4284.388	205			
5	Regression	755.500	5	151.100	8.564	0.000^f^
	Residual	3528.889	200	17.644		
	Total	4284.388	205			

^a^Dependent Variable: Cancer Worry Scale, ^b^Predictors: (Constant). Benefits of Pap Smear Tests, ^c^Predictors: (Constant). Benefits of Pap Smear Tests, Barriers to Pap Smear Test, ^d^Predictors: (Constant). Benefits of Pap Smear Tests, Barriers to Pap Smear Test, Perceived Seriousness of Cervical Cancer; ^e^Predictors: (Constant). Benefits of Pap Smear Tests, Barriers to Pap Smear Test, Perceived Seriousness of Cervical Cancer, Susceptibility to Cervical Cancer, ^f^Predictors: (Constant). Benefits of Pap Smear Tests, Barriers to Pap Smear Test, Perceived Seriousness of Cervical Cancer, Susceptibility to Cervical Cancer, Health Motivation

Including benefits of pap smear tests in the model did not result in a statistically significant change in *R*^2^ (0.004) (*p* = 0.343). However, the addition of barriers to pap smear test led to a statistically significant change in *R*^2^ by 0.031 (*p* = 0.012), and the inclusion of perceived seriousness of cervical cancer further increased the *R*^2^ value by 0.126, which was statistically significant (*p* = 0.000). Subsequently, adding susceptibility to cervical cancer and health motivation individually to the model did not result in a statistically significant change in *R*^2^ (*p* = 0.060, *p* = 0.655, respectively) (Table 3).

**Table 3:** Model Summary

Model summary									
Model	*R*	*R* square	Adjusted *R* square	Std. Error of the Estimate	Change statistics				
					*R* square change	*F* change	df1	df2	Sig. *F* change
1	0.066^a^	0.004	0.000	4.57268	0.004	0.902	1	204	0.343
2	0.187^b^	0.035	0.025	4.51309	0.031	6.423	1	203	0.012*
3	0.401^c^	0.161	0.148	4.21893	0.126	30.294	1	202	0.000*
4	0.419^d^	0.176	0.159	4.19216	0.015	3.589	1	201	0.060
5	0.420^e^	0.176	0.156	4.20053	0.001	0.200	1	200	0.655

^a^Predictors: (Constant). Benefits of Pap Smear Tests, ^b^Predictors: (Constant). Benefits of Pap Smear Tests, Barriers to Pap Smear Test, ^c^Predictors: (Constant). Benefits of Pap Smear Tests, Barriers to Pap Smear Test, Perceived Seriousness of Cervical Cancer, ^d^Predictors: (Constant). Benefits of Pap Smear Tests, Barriers to Pap Smear Test, Perceived Seriousness of Cervical Cancer, Susceptibility to Cervical Cancer, ^e^Predictors: (Constant). Benefits of Pap Smear Tests, Barriers to Pap Smear Test, Perceived Seriousness of Cervical Cancer, Susceptibility to Cervical Cancer, Health Motivation, ^f^Dependent Variable: Cancer Worry Scale

^*^p ≤ 0.05

A one-unit increase in perceived barriers was found to result in a statistically significant 0.187-unit increase in cancer anxiety, while a change in perceived seriousness of cervical cancer led to a statistically significant 0.322-unit increase in cancer anxiety. Additionally, the final model, which incorporated all concepts of the Health Belief Model Scale for Cervical Cancer and the Pap Smear Test, explained 15.6% of the variance in cancer worry based on the independent variables (Table 4).

**Table 4:** Coefficient values of models

Model		Unstandardized coefficients		Standardized coefficients	*t*	Sig	95.0% confidence interval for B	
		B	Std. Error	Beta			Lower bound	Upper bound
1	(Constant)	13.309	1.980		6.721	0.000	9.405	17.213
	Benefits of Pap Smear tests	0.055	0.058	0.066	0.950	0.343	− 0.059	0.169
2	(Constant)	8.293	2.781		2.982	0.003	2.809	13.777
	Benefits of Pap Smear tests	0.109	0.061	0.132	1.793	0.074	− 0.011	0.230
	Barriers to Pap Smear test	0.104	0.041	0.187	2.534	0.012*	0.023	0.185
3	(Constant)	6.978	2.611		2.672	0.008	1.829	12.126
	Benefits of Pap Smear tests	0.016	0.060	0.019	0.266	0.791	− 0.102	0.133
	Barriers to Pap Smear test	0.046	0.040	0.082	1.152	0.251	− 0.033	0.124
	Perceived seriousness of cervical cancer	0.288	0.052	0.376	5.504	0.000*	0.185	0.391
4	(Constant)	5.718	2.678		2.135	0.034	0.436	10.999
	Benefits of Pap Smear tests	0.026	0.059	0.032	0.439	0.661	− 0.091	0.143
	Barriers to Pap Smear test	0.038	0.040	0.069	0.965	0.336	− 0.040	0.116
	Perceived seriousness of cervical cancer	0.249	0.056	0.325	4.463	0.000*	0.139	0.360
	Susceptibility to cervical cancer	0.268	0.141	0.133	1.894	0.060	− 0.011	0.547
5	(Constant)	5.940	2.729		2.176	0.031	0.558	11.322
	Benefits of Pap Smear tests	0.042	0.069	0.051	0.606	0.545	− 0.095	0.179
	Barriers to Pap Smear test	0.037	0.040	0.067	0.942	0.347	− 0.041	0.116
	Perceived seriousness of cervical cancer	0.247	0.056	0.322	4.379	0.00*	0.136	0.358
	Susceptibility to cervical cancer	0.273	0.142	0.135	1.918	0.056	− 0.008	0.553
	Health motivation	− 0.065	0.145	− 0.035	− 0.447	0.655	− 0.351	0.221

^*^p ≤ 0.05

## Discussion

The current study revealed that perceptions of seriousness and barriers affect fear of cancer levels, except of perceived benefits, health motivation, and perception susceptibility dimensions of Health Belief Model. This finding is interesting which may emphasize the deeply effects of negative perceptions on the fear of cancer. Perceived seriousness encompasses the negative outcomes an individual links to being diagnosed with cancer, associated with future occurrences or existing conditions [23]. Frameworks like the Extended Parallel Process Model [29] and Protection Motivation Theory [30] propose that perceived severity positively relates to fear. In the case of cancer, the potential negative consequences (i.e., threat) can be caused by (1) the disease itself and (2) potential harmful side effects that come with its treatment. Numerous studies have indicated that the perceived severity of a disease and the side effects of its treatment are strong predictors of fear [24, 31, 32]. While assessments of disease threats contribute to fear, factors such as the way in which cancer is linguistically framed also influence this emotion. Public discussions, media representations, and societal perceptions can amplify fear, at times overshadowing objective evaluations [24].

Increased perceived severity has been connected to enhanced information-seeking behavior [33]. However, excessive media consumption has been linked to negative health perception and heightened fear of cancer [34]. The media presents varied narratives about cancer, potentially influencing fear levels. Some studies indicate a positive relationship between television news exposure and increased fear of cancer [35, 34]. Consequently, it is essential to organize public health initiatives that aim to reduce the stigma associated with cancer and to communicate information in a positive and educational manner without inducing fear. In addition, media literacy programs should be established to assist individuals in critically assessing cancer-related news reports. Cultural factors also shape may different reasons for cancer fear, such as Lebanese-American women expressed concern about job loss, while Lebanese women feared saddening families, losing friends, neglecting children, death, suffering, appearance changes, rumors, gossip, and sexual disturbances [36]. These findings highlight the importance of integrating cultural considerations into cancer care and education. Addressing culture-specific beliefs and concerns is essential to alleviate fears and enhance participation in cancer prevention and treatment. Additionally, it is recommended to provide culturally appropriate counseling and adapt cancer awareness initiatives to suit the needs of diverse communities.

According to the Health Belief Model, people are more likely to adopt health behaviors when they perceive the benefits to outweigh the barriers [23]. This study found that not perceive benefits, but perceived obstacles significantly influenced cancer fear levels. These obstacles include monetary expenses, discomfort, insufficient screening knowledge, diagnosis anxiety, and practical issues, such as time constraints and healthcare access [37]. Emotional barriers, particularly “feeling scared,” are the most frequently cited impediments [38]. In low- and middle-income countries, cancer is often linked to higher mortality and limited treatment options, intensifying fear and reluctance to seek medical attention [39, 40]. This finding corresponds with findings suggesting that populations with more negative cancer beliefs tend to experience stronger fear-related barriers than those in high-income settings [41, 42]. However, an enhanced public understanding of cancer risk factors, symptoms, and treatment options can help alleviate these fears and barriers [43]. Research indicates that individuals with greater cancer awareness report fewer practical obstacles in seeking medical advice [44]. Therefore, public awareness campaigns and school- or community-based interventions may be effective in reducing fear and encouraging early screening. Although funding for such programs is often scarce in low- and middle-income countries, investing in cancer education may ultimately be cost-effective by reducing morbidity and mortality.

Research findings indicate that women who are employed and live in nuclear families generally experience a reduced fear of cancer. This correlation may be attributed to the non-traditional roles these women assume, potentially providing them with greater independence and authority over their lives than those in more conventional family structures. Both internal and external gender-based discrimination often restrict healthcare access and can intensify cancer-related fears, particularly among women who lack control over health-related decisions [45]. The notion of self-care agency is fundamental to addressing fear and cancer-related concerns. Self-care management practices suggest that women with higher levels of agency, such as the capacity to embrace a healthy lifestyle, track their symptoms, and pursue treatment, may exhibit lower levels of fear of cancer [46]. In communities where women exercise more control over their lives and health, as is often the case in nuclear families or professional environments, their self-efficacy and ability to manage health issues are likely to be enhanced, resulting in diminished fear of cancer [47].

### Strengths and limitations

The present study had some limitations. The study sampled women who applied one healthy life center which is in the city center. However, it also includes the heterogeneity of women’s characteristics such age, education level, employment status, and perception economy levels.

## Conclusion

The study revealed that perceived severity of cervical cancer, and perceived barriers for cervical cancer and Pap Smear test effect fear of cancer levels of women who applied to cervical cancer screening program. Due to meanings of cancer may lead to perceived severity, and barriers. Also, women who employ and nuclear family type have less fear of cancer levels. This correlation may be attributed to the non-traditional roles these women assume, potentially providing them with greater independence and authority over their lives than those in more conventional family structures.

## Data Availability

The data underlying this article cannot be shared publicly due to the privacy of individuals that participated in the study. The data will be shared on reasonable request to the corresponding author.
